# Scarlet Fever Convalescents

**Published:** 1887-12-24

**Authors:** W. E. Gladstone


### Scarlet Fever Convalescents.

By the Right Hon. W. E. Gladstone, M.P.

I see no reason to change the sentiments expressed in my speech on behalf of the Mary Wardell Convalescent Home for Scarlet Fever; therefore, if it will be of any use in The Hospital, you are most welcome to publish it. I require an apology for undertaking to speak upon a subject where, after all, we move under the shield and protection of medical authority. Without that, at least, it would not have been easy to step forward with confidence, but I rejoice to think that, if there have been differences of opinion upon the subject of providing in separate buildings for infectious convalescents, they are now so completely absorbed in an overwhelming consent of those most competent to judge, nearly, I believe, without exception, that we may feel justified in giving free scope to all those sentiments upon a subject of this kind, which, as unscientific persons, I think, we cannot help entertaining. I am persuaded that the purpose of this movement to establish homes for persons convalescent from scarlet fever has a perfectly legitimate place in the general philanthropic movements of the day. It has been reserved for this generation to supply—to use a phrase now familiarly known to us—a missing link in the public provision made for the treatment of disease when it selects for its victims those who have not the command from their own resources of the best means of meeting it.

**A Contemporaneous Experience.**

The time of my own residence in London covers, I believe, the whole period since the very first efforts were made to found anything in the nature of a convalescent hospital or retreat. I believe what is known as the House of Charity, now very usefully employed for different purposes, was either absolutely the first, or among the first—perhaps a rude and imperfect effort—to put some new provision of care and sympathy into the space which separated the period of acute suffering and substantive indisposition in a hospital, from the period of perfect recovery in which alone persons of the labouring classes could with safety and with satisfaction resume their usual employment. This process has gone forward amid general satisfaction, and, I believe, with a conviction upon the part of all who have witnessed it, that there never was a case in which a want more glaring was supplied by means more appropriate. Now, under those circumstances, one would require what is called the burden of proof to be thrown upon those who should contend for the opinion that the particular disease of scarlatina, as it appears to be now technically called, including scarlet fever, should not have the benefit of arrangements of that kind.

**The Virulence of Scarlet Fever.**

I should have thought, speaking in the character of an ignorant person, that there were some special reasons applying to this disease, perhaps more than to any other, why such a provision should be made for it. The later stages of the disease—what are commonly called the "dregs" of this disease—as many of us have known them, even though not professionally connected with the subject, are of peculiar subtlety and peculiar violence. Possibly some of those who now read this may, like myself, belong to a family in which this very cause led ultimately to a fatal result in a much-beloved member of it; but, I believe, there is no doubt that those very causes and agencies which are at work after the acute stage of the disease has passed by, have a peculiar virulence and a peculiarly formidable character in the case of scarlet fever. Then, again, if we look to the magnitude of the subject, it is very easily presented in a few figures when we learn that there are no fewer than an estimated number of about twenty-five thousand cases of this disease in the metropolis in the course of a year. I have borrowed from an excellent production of Sir Rutherford Alcock an account of the exact number of the deaths that occurred from this cause in certain years. In 1877 the deaths in London alone amounted to 1,576; in 1878 there were 1,792; in 1879, 2,706; in 1880, 3,073.

**Valuable and Original.**

Perhaps the most valuable and certainly the only original contribution that in these remarks I shall have made to the knowledge of those interested, will be found in the fact which I have learnt from the Registrar General's Office. He acquaints me that in the year 1881, although there was a mitigation as compared with the previous year, yet the deaths amounted to 2,371, a very large proportion of those deaths being of children under five years of age. In 1882 there were 2,006 deaths; in 1884, 1,430; in 1885, 722; and in 1886, 686. It may assist to deepen the popular impression concerning the magnitude and importance of the subject, if I state that in the last 15 years (1872-86) it appears that the deaths from scarlatina in the metropolis have averaged 1,887 per annum, while in the same 15 years the deaths due to that most formidable scourge of society whose name is familiar to us all, smallpox, have averaged considerably less, or 883 per annum. Whether we consider the magnitude of the subject or the propriety of the object, or the known and experienced utility of the description of institution which I desire to advocate and to help to promote; I think that the case is one which is sound in all its parts, and which can require, at any rate from those who can speak with greater authority than myself, no lengthened exposition to commend it to the warmest support of all classes of the people.

